# Tumor Microenvironment: Implications in Melanoma Resistance to Targeted Therapy and Immunotherapy

**DOI:** 10.3390/cancers12102870

**Published:** 2020-10-06

**Authors:** Italia Falcone, Fabiana Conciatori, Chiara Bazzichetto, Gianluigi Ferretti, Francesco Cognetti, Ludovica Ciuffreda, Michele Milella

**Affiliations:** 1 Medical Oncology, IRCCS—Regina Elena National Cancer Institute, 00144 Rome, Italy; fabiana.conciatori@ifo.gov.it (F.C.); chiara.bazzichetto@ifo.gov.it (C.B.); gianluigi.ferretti@ifo.gov.it (G.F.); francesco.cognetti@ifo.gov.it (F.C.); 2 SAFU, Department of Research, Advanced Diagnostics, and Technological Innovation, IRCCS—Regina Elena National Cancer Institute, 00144 Rome, Italy; ludovica.ciuffreda@ifo.gov.it; 3 Section of Oncology, Department of Medicine, University of Verona School of Medicine and Verona University Hospital Trust, 37126 Verona, Italy; michele.milella@univr.it

**Keywords:** melanoma, targeted therapy, immunotherapy, tumor microenvironment, therapeutic resistance

## Abstract

**Simple Summary:**

The response to pharmacological treatments is deeply influenced by the tight interactions between the tumor cells and the microenvironment. In this review we describe, for melanoma, the most important mechanisms of resistance to targeted therapy and immunotherapy mediated by the components of the microenvironment. In addition, we briefly describe the most recent therapeutic advances for this pathology. The knowledge of molecular mechanisms, which are underlying of drug resistance, is fundamental for the development of new therapeutic approaches for the treatment of melanoma patients.

**Abstract:**

Antitumor therapies have made great strides in recent decades. Chemotherapy, aggressive and unable to discriminate cancer from healthy cells, has given way to personalized treatments that, recognizing and blocking specific molecular targets, have paved the way for targeted and effective therapies. Melanoma was one of the first tumor types to benefit from this new care frontier by introducing specific inhibitors for v-Raf murine sarcoma viral oncogene homolog B (BRAF), mitogen-activated protein kinase kinase (MEK), v-kit Hardy–Zuckerman 4 feline sarcoma viral oncogene homolog (KIT), and, recently, immunotherapy. However, despite the progress made in the melanoma treatment, primary and/or acquired drug resistance remains an unresolved problem. The molecular dynamics that promote this phenomenon are very complex but several studies have shown that the tumor microenvironment (TME) plays, certainly, a key role. In this review, we will describe the new melanoma treatment approaches and we will analyze the mechanisms by which TME promotes resistance to targeted therapy and immunotherapy.

## 1. Introduction

Melanoma is one of most aggressive human tumors, arising from the uncontrolled proliferation of melanocytes, the skin cells responsible for the production of melanin. In terms of incidence, malignant melanoma accounts for approximately 5% of all malignant tumors and its incidence is highly variable, depending on race and geographical variations: It is predominantly diagnosed in Caucasians and 85% of cases occur in North America, Europe, and Oceania. Although highly curable when diagnosed in an early phase, melanoma is an aggressive disease with five years’ relative survival of only 25%, when diagnosed at an advanced metastatic stage [1,2]. Like many other solid tumors, malignant melanoma is highly heterogeneous and substantially resistant to unselective treatments, such as chemotherapy. In the past few years, mutational analysis and next-generation sequencing (NGS) approaches have shown that somatic mutations in *BRAF* or neuroblastoma RAS viral oncogene homolog (*NRAS*) genes promote deregulated survival and migration when combined with genetic alterations and/or epigenetic events that support senescence bypass [3,4].

Moreover, metastatic melanoma is considered a perfect example of immunogenic tumor because it is characterized by the consistent presence of lymphocid infiltrate, as compared to other cancers [5]. Based on these observations, molecularly targeted therapy and immunotherapy have revolutionized the approach to melanoma treatment and overall management. Clinical evidence has shown extremely encouraging results in terms of overall survival (OS) in patients treated with targeted therapy and immunotherapy [6,7,8,9]. Despite many important advances, however, development of the resistance remains a significant obstacle to melanoma curability and can be modulated by several factors, both intrinsic and extrinsic to the cancer cell. One such important factor is certainly the tumor microenvironment (TME), an intricate and complex network of cells, molecules, and paracrine factors that are tightly interconnected with melanoma cells, thereby influencing their initiation, progression, and sensitivity/resistance to therapeutic interventions. 

In this review, we focused on melanoma microenvironment, analyzing its implications in therapy resistance.

## 2. Melanoma Targeted Therapy and Immunotherapy: An Overview

**Figure 1:**
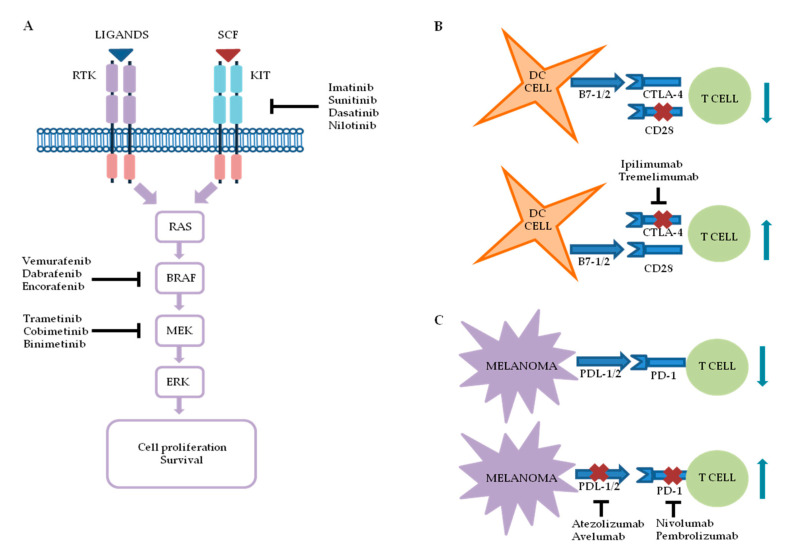
Schematic representation of the most important approaches to melanoma treatment. (**A**) Mitogen-activated protein kinase (MAPK) signaling is often deregulated in melanoma and, for this reason, several drugs against the components of the pathway have been developed. Although less frequently mutated, the v-kit Hardy–Zuckerman 4 feline sarcoma viral oncogene homolog (KIT) receptor tyrosine kinase, represents another molecular target for certain melanomas, and KIT inhibitors can be used in combination with chemotherapy or immunotherapy. (**B**) Cytotoxic T-lymphocyte-associated protein 4 (CTLA-4), interacting with B7 ligands present on the surface of dendritic cells (DCs), prevents the activation of T lymphocytes. The functional block of CTLA-4 mediated by monoclonal antibodies supports the interaction between B7 ligands and CD28, positive regulator receptor of the T lymphocyte activity. (**C**) Melanoma cells usually express elevated levels of programmed death ligand (PDL) 1 and 2, which, through interaction with programmed cell death protein 1 (PD-1) receptor on T cells, block their activation. Pharmacological inhibition of the PD-1 axis restores T cells’ ability to recognize and kill tumor cells.

The identification of new molecular targets and the availability of modern immunotherapeutic approaches have revolutionized the treatment of advanced melanoma. By enabling the detection of genomic, transcriptional and epigenetic changes, NGS has allowed the identification of specific targets, thereby allowing for the development and optimization of treatments interfering with specific molecular targets [10,11]. Although such novel therapeutic approaches have demonstrated clinical efficacy and rapid responses in most patients, acquired resistance represents a significant challenge and has yet to be overcome. The most important therapeutic approaches to contemporary melanoma treatments are summarized in Figure 1.

### 2.1. Targeted Therapy

#### 2.1.1. BRAF Inhibitors

The Mitogen-activated protein kinase (MAPK) pathway represents the signaling pathway most frequently dysregulated in melanoma and many inhibitors against this cascade have been developed at preclinical and clinical levels [12,13]. 

The serine/threonine protein kinase BRAF, physiologically involved in the control of cellular growth, is mutated in about 50% of all melanomas [14]. Although 20 individual BRAF mutations have been described, approximately 90% of BRAF-mutant melanomas present a mutation leading to the substitution of the valine 600 residue by glutamic acid (V600E) [15]. BRAF mutation per se is not sufficient to promote melanoma formation, but several studies have highlighted a crucial role of the mutant protein in disease progression [16]. In experiments in vitro, BRAF inhibition blocks melanoma growth and stimulates apoptosis, while, in vivo, reduced tumor formation is observed in mouse models [15,16,17,18,19,20]. Several BRAF inhibitors have been developed and approved for the treatment of melanoma and other BRAF-mutant tumors. Vemurafenib (or PLX4032) and dabrafenib (or GSK2118436) are potent and selective BRAF kinase inhibitors approved by the Food and Drug Administration (FDA) in 2011 and 2013, respectively, for metastatic melanoma. They shut down signaling through extracellular signal-regulated kinase (ERK) and, consequently, inhibit cellular growth in BRAF-mutant melanoma cells and induce tumor regression in xenograft models [21,22,23]. To date, these two drugs are employed, alone or in combination with chemotherapy, other targeted agents and immunotherapy in many clinical trials because they have shown to induce a substantial clinical responses and to prolong OS and progression-free survival (PFS) in melanoma patients [24,25]. Encorafenib (or LGX818) is a relatively novel BRAF inhibitor, approved by the FDA in June 2018. At preclinical level, it inhibits BRAF^V600E^ kinase activity determining cell growth inhibition in vitro and tumor regression in vivo in mouse models of BRAF-mutant melanoma [26]. A recent clinical trial, conducted on 577 BRAF-mutant melanoma patients, showed that encorafenib alone or in combination with binimetinib (MEK inhibitor) brings benefits, in terms of survival and tolerability, as compared to treatment with vemurafenib alone [7]. 

It is now well known that BRAF inhibition alone in melanoma can be overcome by many different mechanisms of resistance, some of which encompass downstream reactivation of MAPK pathway [27]. This phenomenon, called “paradoxical effect”, is determined by dimerization between wild-type (wt) or kinase-inhibited BRAF and v-raf1 murine leukemia viral oncogene homolog 1 (CRAF), causing RAF signaling reactivation [28,29]. Even in tumor models different from melanoma, such as BRAF- wt/RAS-mutant (mut) lung and pancreatic cancers, we confirmed paradoxical MAPK reactivation upon pharmacological BRAF kinase inhibition; however, simultaneous treatment with MEK inhibitors switched MAPK off again and induced a synergistic reduction of cell growth both in vitro and in vivo [30]. Indeed, in the past few years, clinical treatment of BRAF-mutant melanoma patients shifted toward a vertical combination of BRAF and MEK inhibitors, able to determine major improvements in terms of OS and PFS [8,30,31,32,33].

#### 2.1.2. Mitogen-Activated Protein Kinase Kinase (MEK) Inhibitors

Trametinib (GSK1120212), cobimetinib (GDC-0973), and binimetinib (MEK162) are all potent selective inhibitors of MEK1 and MEK2 and are associated with significant cell growth inhibition in in vitro experiments and antitumor activity in mouse models of BRAF-mutant melanoma [34]. As described above, they are used as either monotherapy or, more often, in combination with dabrafenib, vemurafenib, and encorafenib, respectively, in patients affected by BRAF-mutant advanced melanoma [15,35,36]. MEK inhibitors also determine modest benefits in terms of PFS for melanoma patients whose tumors carry missense mutations in *NRAS* (occurring in about 20% of melanoma cases) [37,38,39,40,41]. 

#### 2.1.3. V-kit Hardy–Zuckerman 4 Feline Sarcoma Viral Oncogene Homolog (KIT) Inhibitors

Activating somatic mutations in the *KIT* proto-oncogene are found in approximately 2–8% of melanomas, especially in those arising in mucosal and acral localizations (10–20% of the cases, respectively) [42,43]. When *KIT* is mutated, in exons 11 and 13, the regular growth and differentiation of melanocytes becomes uncontrolled; moreover, these mutations are generally mutually exclusive with the more frequent ones, such as those in *BRAF* and *NRAS* [13,44]. Many inhibitors, developed to block KIT and other tyrosine kinase receptors (RTKs), were analyzed in different clinical trials for melanoma such as imatinib, sunitinib, dasatinib, and nilotinib in combination with chemotherapy and immunotherapy [45,46].

### 2.2. Immunotherapy

Given its immunogenic characteristics, melanoma has been one of the solid tumors in which immunotherapy, using many different strategies aimed at stimulating the patient’s immune system to recognize and eliminate cancer cells, has been most intensively studied [5]. Current immunotherapy approaches to human malignant melanoma include: monoclonal antibodies against immune checkpoint (ICIs), T-cell therapy, and cancer vaccines. Monoclonal antibodies inhibiting specific ICIs, including anti-programmed cell death protein 1 (PD-1), anti-programmed death ligand-1 (PDL-1), and cytotoxic T-lymphocyte-associated protein 4 (CTLA-4), alone or in combination, have been tested with great success in clinical trials and approved by the FDA for the treatment of advanced melanoma [47,48]. 

#### 2.2.1. Anti-CTLA-4

CTLA-4, present on the surface of cluster differentiation (CD) 4^+^ and CD8^+^ lymphocytes, is another important pharmacological target for the treatment of several neoplastic forms, including metastatic melanoma [49]. Upon binding to the B7-1 (CD-80) and B7-2 (CD86) ligands on dendritic cells (DCs), CTLA-4 prevents their binding to the CD28 co-stimulatory receptor, which positively regulates lymphocyte activity, thereby triggering inhibitory signals that negatively regulate T-lymphocyte activation. Unlike the PD-1 axis (see below), which operates during the effector phase of the immune response, CTLA-4 and its inhibitors are implicated during the early stages of antigen presentation, leading to the first activation of T cells and immune recognition of the tumor. This prerogative is one of the reasons why combined checkpoint inhibition (with anti-CTLA-4 and anti-PD-1 agents) results in synergistic antitumor efficacy in the clinical setting [50]. Ipilimumab (MDX-010) is a humanized antibody against CTLA-4, currently approved by the FDA for the treatment of metastatic melanoma, either alone or in combination with PD-1 inhibitors. Ipilimumab significantly improved OS, as compared to cytotoxic chemotherapy, in metastatic melanoma, resulting in a proportion of patients experiencing prolonged disease control and causing a plateau in the survival curve at three years [51,52,53]. Tremelimumab (CP-675,206) is another monoclonal antibody against CTLA-4, which promotes important and durable tumor regressions in approximately 10% of metastatic melanoma patients; however, unlike ipilimumab, no significant changes in terms of survival were observed between patients treated with tremelimumab and those treated with chemotherapy [54]. Both of the two CTLA-4 antibodies are currently being studied in over 300 clinical trials involving patients with malignant melanoma [45].

#### 2.2.2. Anti-PD-1

The PD-1 receptor, expressed on the surface of several immune cells, physiologically inhibits T cell activity upon binding to its ligands PDL-1 and -2. Activation of the PD-1/PDL-1/2 axis is frequently used by cancer cells to escape immune-mediated killing, often through suppression of downstream effectors of the phosphatidylinositol 3-kinase (PI3K) pathway and cell cycle arrest in cytotoxic lymphocytes (CTL) [55]. Melanoma is generally characterized by high levels of PDL-1 expression, which correlates with poor prognosis; based on this finding, several monoclonal antibodies directed against the PD-1 axis have been developed and are used for melanoma treatment [56,57,58,59,60]. Nivolumab (BMS-936558, MDX-1106) and pembrolizumab (MK-3475) represent the two most important monoclonal antibodies against PD-1. They positively regulate the reactivation of T cells by blocking the interaction between the PD-1 receptor and its ligands, and have been studied in clinical trials, either alone or in combination with other ICIs, such as ipilimumab (CTLA-4 inhibitor, see above), chemotherapy, and targeted therapy. Preclinical studies have shown impressive results in terms of tumor growth inhibition; most importantly, clinical studies conducted in metastatic melanoma patients confirmed a clinically and statistically significant impact of these agents in terms of PFS and OS prolongation [21,61,62,63,64,65]. The phase III clinical trial CheckMate 067, completed in 2015, has shown a significant survival benefit (in terms of both PFS and OS) for metastatic melanoma patients treated with nivolumab, either alone or combined with ipilimumab. Compared with ipilimumab monotherapy the risk of death was reduced by 48% (*p* < 0.001) by the combination of nivolumab plus ipilimumab and by 36% (*p* < 0.001) by nivolumab alone [66,67]. Pembrolizumab revolutionized the treatment of patients with advanced melanoma versus ipilimumab, significantly improving PFS and OS [68]. Recently, the phase III clinical study KEYNOTE-006 further confirmed this finding, showing the superiority of pembrolizumab even after five years of follow-up. The median OS was 32.7 months for patients treated with pembrolizumab and 15.9 months for groups of patients treated with ipilimumab. The PFS was 8.4 months and 3.4 months for patients treated with pembrolizumab and ipilimumab, respectively [65,69]. Several PDL-1 inhibitors are now involved in clinical trials for melanoma. Atezolizumab has shown promising results in monotherapy for patients with metastatic melanoma [70]. Recently, the results of a triple combination of atezolizumab, vemurafenib, and cobimetinib, in patients with BRAF-mutant melanoma, were reported: the phase III clinical study IMspire 150 showed a significant PFS benefit (15.1 vs. 10.6 months) for patients treated with the triple combination, as compared to those who received vemurafenib and cobimetinib only [71]. Avelumab is another human anti-PDL-1 antibody involved in a phase I clinical trial (JAVELIN) for previously treated metastatic melanoma patients. The trial showed long-lasting and clinically meaningful disease control, with promising PFS and OS duration [72].

#### 2.2.3. Alternative Melanoma Immunotherapies

Immunological therapies for melanoma are not limited to the use of checkpoint inhibitors. Isolation and ex vivo expansion of tumor-infiltrating lymphocytes (TILs) and their reintroduction in patients subjected to surgical removal of melanoma lesions has shown potential benefits; however, such complex approach remains limited to a research setting and is available only in a few specialized centers [73]. Chimeric antigen receptor T-cells (CAR-T) are another therapeutic approach that involves the use of engineered T cells to promote their ability to recognize cancer cells. Although this therapeutic field has made great strides and achieved unique results in the treatment of hematological malignancies, it has not produced the same positive effects in melanoma and other solid tumors, due to the presence of a highly immunosuppressive microenvironment in these contexts [74]. New treatment scenarios involve the development of vaccines that can stimulate the patient’s immune system against tumor-associated antigens. Although preclinical research has obtained promising results in several cancer models, including melanoma, clinical results to date have been largely unsatisfactory [75,76]. Glycoprotein (Gp)-100, as an example, is a synthetic peptide encompassing a few amino acid residues of the trans-membrane Gp-100 protein expressed by melanoma cells. It has been formulated into a vaccine developed for advanced melanoma and utilized in clinical trials [77]. Vaccination with this peptide stimulates the host immune system inducing a CTL response that recognizes and kills melanoma cells in vitro. In a clinical study conducted in 185 patients with advanced melanoma, the immune-stimulating cytokine interleukin (IL)-2 was given alone or in combination with the Gp-110 peptide; patients treated with this combination presented a significant increase in terms of OS [78]. Vitespen is another vaccine developed for melanoma treatment, encompassing a heat shock protein/peptide complex (Gp-96) obtained and purified from surgically excised tumors. In advanced melanoma it has failed to show survival benefits but continues to stimulate the attention of researchers due to its extremely favorable toxicity profile [79,80].

## 3. TME Implications in Drug Resistance for Melanoma

**Figure 2:**
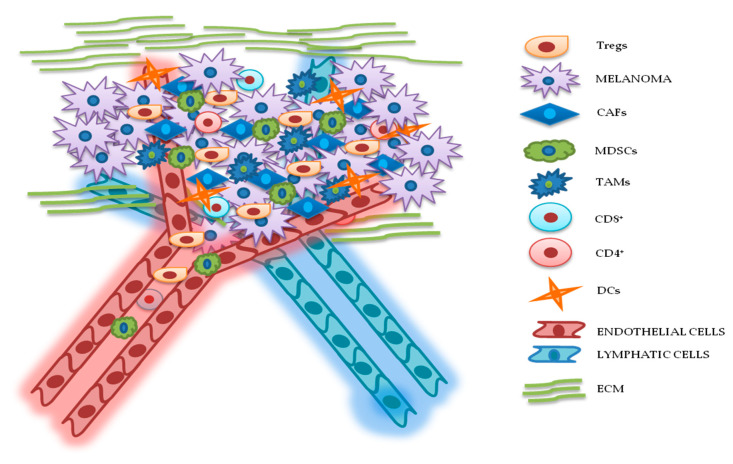
Relationship between melanoma and tumor microenvironment (TME). In this figure, is illustrated schematically the reciprocal interactions between melanoma cells and the other components of TME. Melanoma’s TME, involved in tumor growth, progression, and drug resistance, is essentially represented by regulatory T cells (Tregs), cancer-associated fibroblasts (CAFs), myeloid-derived suppressor cells (MDSCs), tumor-associated macrophages (TAMs), cluster differentiation (CD) 4^+^/CD8^+^ lymphocytes, dendritic cells (DCs), endothelial and lymphatic cells, and extracellular matrix (ECM).

Development of therapeutic resistance arguably represents the most important challenge in cancer therapy. Such phenomenon is associated with disease progression and low survival rates and is promoted by the ability of cancer cells to activate both intrinsic (i.e., dependent on genetic changes occurring in the cancer cell itself) and extrinsic (i.e., mediated by cross-talk mechanisms occurring between cancerous and noncancerous cells) escape mechanisms. Tumors are characterized by high genomic instability and heterogeneity and these prerogatives may lead to both primary (or de novo) or acquired resistance (i.e., occurring in cells previously responsive to the same treatment). Most importantly, the selective pressure applied by treatment itself may select out specific mechanisms of resistance. In some instances, therapeutic resistance occurs independent of genetic changes modifying cancer cells’ acquired capabilities: In these cases, the insurgence of drug resistance can be attributed to changes occurring in different compartments of the TME. Indeed, tumor masses, including those that form in metastatic melanoma, should not be considered as isolated contexts without interactions; indeed, it has long been known that tumor cells "cross talk" continuously with many cellular and acellular components of the tumor stroma, which surrounds and penetrates the tumor mass. Such intricate structures constitute the TME and are characterized by mutual and continuous interactions between tumor and nontumor cells. TME is composed of cells and extracellular components of different origins, which contribute in several ways to the various stages of tumor progression (Figure 2) [81,82].

### 3.1. Cellular Components

#### 3.1.1. Cancer-Associated Fibroblasts (CAFs)

**Figure 3:**
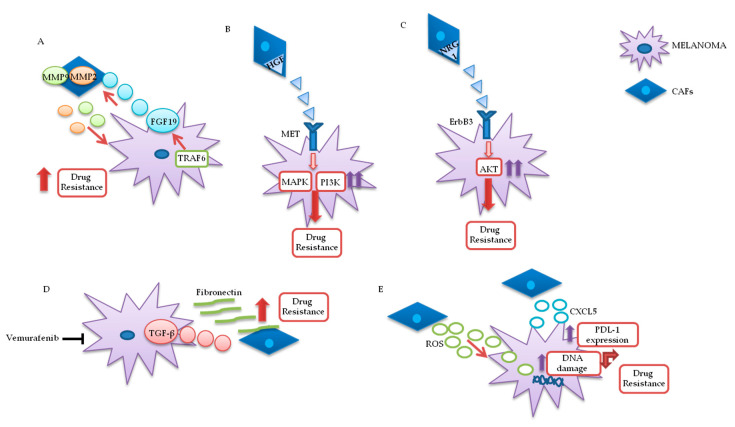
Melanoma/CAFs’ paracrine interconnections. (**A**–**E**) In this figure, is illustrated schematically the mutual interactions between melanoma cells and cancer-associated fibroblasts (CAFs). Several factors are implicated in these intricate interconnections at the basis of drugs resistance: Tumor necrosis factor receptor-associated factor 6 (TRAF6), fibroblast growth factor 19 (FGF19), metalloproteinases 2 and 9 (MMP2 and MMP9), hepatocyte growth factor (HGF), neuregulin 1 (NRG1), V-erb-b2 avian erythroblastic leukemia viral oncogene homolog3 (ErbB3), transforming growth factor β (TGF-β), reactive oxygen species (ROS), CXC motif chemokine 5 (CXCL5), programmed death-ligand 1 (PDL-1).

Fibroblasts are important components of the stroma and their physiological functions encompass synthesis of extracellular matrix (ECM) and regulation of the inflammatory process. Upon tight (direct or mediated by soluble factors) interaction with cancer cells, fibroblasts differentiate into CAFs, which are characterized by specific markers: α smooth muscle actin (α-SMA), fibroblast activation protein (FAP), vimentin, fibroblast specific protein 1 (FSP1), and platelet-derived growth factor receptor (PDGFR)-α and β [83,84,85]. CAFs are involved in many cellular processes, including ECM remodeling, angiogenesis, and cell-to-cell interactions; in vivo, their activation is fundamental for tumor neo-vascularization [86,87]. In vitro and in vivo studies have shown that the continuous and persistent interactions between tumor cells and CAFs promote many aspects of the tumorigenic process, such as tumor progression, metastasis, and drug resistance. Melanoma cells, co-cultured with CAFs or grown in their conditioned media, display greater invasion and migration capabilities, as compared to the same cells cultured in isolation [88,89]. Recent studies also confirm that CAFs’ activation is probably a crucial step for melanoma metastasis formation. Indeed, mice, in which the CAFs are inhibited by β-catenin suppression, displayed markedly decreased tumor-mediated vascularization [87,90]. CAFs and tumor cells reciprocally influence each other’s biological behavior, and such cross talk is finely regulated by specific molecular mechanisms. As described in Figure 3A, the co-regulation system can involve tumor necrosis factor receptor-associated factor 6 (TRAF6), expressed in CAFs’ activated and melanoma cells [91]. 

In melanoma cells, TRAF6 promotes nuclear factor kappa-light-chain-enhancer of activated B cells (NFkB)-dependent release of fibroblast growth factor 19 (FGF19), implicated in the transformation and activation of fibroblasts. FGF19-mediated CAFs’ activation supports, in turn, the malignant and invasive phenotype of melanoma cells and their drugs resistance. On the other hand, TRAF6 upregulation in fibroblasts results in ECM remodeling through the release of matrix metalloproteinases (MMPs) 2 and 9 [91]. 

The mutual interaction between melanoma and CAFs can promote drug resistance in different ways. Straussman and collaborators highlighted the role of hepatocyte growth factor (HGF) in the development of acquired resistance to BRAF inhibitors (Figure 3B). Co-culture systems and proteomics analysis showed that HGF secreted by fibroblasts, by interacting with its mesenchymal ephitelial transition (MET) receptor on melanoma, induced MAPK and PI3K pathways’ activation, thereby promoting resistance to RAF inhibition. Simultaneous downregulation of both RAF and MET reverted resistance in vitro, and it has been proposed as a possible therapeutic approach for the treatment of BRAF-mutant melanomas. Most importantly, the authors confirmed increased HGF expression in stromal cells of BRAF-mutant melanoma patients undergoing BRAF-targeted treatment in vivo, which resulted in poor prognosis and decreased response to treatments [92]. Neuregulin 1 (NRG1) is another paracrine factor through which CAFs may influence melanoma response to MAPK inhibitors (Figure 3C). NRG1 is the ligand of v-erb-b2 avian erythroblastic leukemia viral oncogene homolog3 (ErbB3), which is upregulated in melanoma cells after treatment with BRAF inhibitors. The use of ErbB3/ErbB2 antibodies restores the cytotoxic activity of these drugs in BRAF-mutant melanoma cell lines [93]. Furthermore, vemurafenib treatment increases the production of transforming growth factor β (TGF-β) by melanoma cells; TGF-β, in turn, causes CAFs’ activation and increased fibronectin production, involved in BRAF inhibitors’ resistance (Figure 3D) [94]. 

Moreover, paradoxical MAPK activation, induced by BRAF inhibitors in genetically “normal” stromal cells, promotes a “therapy-resistant” microenvironment: Intravital imaging analyses conducted in melanoma have shown major paradox MAPK reactivation, especially in areas with high stromal density. Such activated CAFs, in turn, promoted matrix remodeling and ERK reactivation in melanoma, through integrin β1/focal adhesion kinase (FAK)/v-src sarcoma (Schmidt–Ruppin A-2) viral oncogene homolog avian (Src) signaling [95].

Uncontrolled production of reactive oxygen species (ROS) by TME fibroblasts is also associated with resistance to BRAF-targeted agents in melanoma (Figure 3E). Aging fibroblasts tend to release high levels of ROS in the TME, thereby modulating MAPK and PI3K pathways’ activation in tumor cells and promoting cells’ growth and drug resistance [96]. Such phenomenon could potentially be reversed by treating melanoma cells with antioxidants, thereby restoring drug responsiveness [97]. Along these lines, a recent preclinical study analyzed the role of secreted frizzled-related protein 2 (sFRP2), a wingless type MMTV integration site family member (Wnt) antagonist, in vemurafenib resistance: The sFRP2, produced and released in the TME by aged fibroblasts, actives a cascade of events in melanoma cells, ultimately leading to the loss of the redox effector apurinic/apyrimidinic endonuclease 1 (APE1). This condition significantly reduces the ability of melanoma cells to overcome ROS-induced DNA damage. Moreover, sFRP2-mediated inhibition of β-catenin leads to reduced melanoma response to vemurafenib [97]. 

It has recently been shown that CAFs are involved in the induction of a protumor immune microenvironment in many cancer models, favoring tumor growth and pharmacological resistance [98,99,100]. CAFs-mediated CXC chemokine ligand 2 (CXCL-2) production promotes regulatory T cells’ (Tregs) growth and recruitment in the tumor stroma [100,101]. Melanoma-associated fibroblasts also directly influence tumor cells’ ability to adapt and modify the response to immunotherapy. Experiments conducted on melanoma cell cultures have shown that CAFs release CXC motif chemokine 5 (CXCL5), which, in turn, induces PI3K/ protein kinase B (AKT)-dependent PDL-1 expression and resistance to immunotherapy in melanoma cells (Figure 3E) [102]. In addition, TGF-β secreted by CAFs also promotes resistance to PD-1 inhibitors: Transcriptomic and flow cytometric analysis, conducted on biopsies from 94 melanoma patients at various treatment stages, revealed a subset of patients characterized by loss of major histocompatibility complex 1 (MCH-I) and disease progression: Such phenomenon, induced by TGF-β released by CAFs, promotes a microphthalmia-associated transcription factor (MITF)_low_/AXL_high_ phenotype in melanoma cells, associated with resistance to MAPK pathway and PD-1 inhibitors [103].

Finally, CAFs’ involvement in drug resistance is not limited to the production of paracrine factors but is also associated with cell-to-cell contact with cancer cells. Several studies have shown that fibroblasts create a physical barrier around the tumor mass and directly activate survival pathways in cancer cells [104,105,106]. In particular, these interactions are promoted by N-cadherin, expressed by both melanoma cells and fibroblasts, and are involved in tumor activation of the survival pathway PI3K/AKT/ BCL2 associated agonist of cell death (BAD) [104].

#### 3.1.2. Lymphocytes

The immuno-microenvironment is characterized by T lymphocytes that recognize antigenic peptides presented by other components of the immune system [107]. CD4^+^ T cells act as immune response “adjuvants” through the secretion of specific cytokines. CD8^+^ T lymphocytes, on the other hand, are responsible for direct antigen/tumor cell individuation/elimination and are considered the most important mediators of tumor immune surveillance [82,108]. 

Depending on their genetic background, melanoma cells can influence the development of an immunosuppressive microenvironment. Phosphatase and tensin homolog deleted on chromosome 10 (*PTEN*), for example, is an important tumor suppressor gene often mutated/deleted in several cancer types, including melanoma; indeed, PTEN loss is present and concomitant with BRAF mutations in about 44% of melanomas and is associated with reduced OS [109]. PTEN loss promotes the formation of TME with low levels of cytotoxic T and natural killer (NK) cells and high concentrations of immunosuppressive elements, such as myeloid-derived suppressor (MDSCs) and Tregs cells [110]. As described in in vitro and in vivo studies, PTEN-null melanoma cells inhibit antitumor activity of T cells and, consequently, response to immunotherapy. Through its negative regulation of PI3K and signal transducer and activator of transcription (STAT) 3 pathways, PTEN inhibits the production of immunosuppressive cytokines, such as IL-6 and 10 and vascular endothelial growth factor (VEGF). In melanoma, PTEN loss promotes STAT3 activation and, consequently, overproduction of these cytokines [111]. Moreover, PTEN loss is associated with reduced T cells’ recruitment to the tumor site and cytotoxic activity [112].

An immunosuppressive TME influences the differentiation of dysfunctional CD8^+^ T lymphocytes, i.e., T cells with reduced growth and effectors’ cell recognition capacity and high concentrations of PD-1 and CTLA-4 receptors. If physiological conditions such as this status are necessary for immune homeostasis and to avoid self-reactive phenomena, in tumor contexts it may be an escape route that cancer cells use to evade immune response and promote resistance to immunotherapy [113]. A study conducted in patients with advanced melanoma demonstrated the presence of a subpopulation of T cells with high levels of PD-1 and immunoglobulin and mucin domain-containing molecule 3 (Tim3), another inhibitory receptor. Tim3 inhibition partially reverted the dysfunctional condition of T cells and increased their antitumor abilities. These results form the rationale for simultaneous blockade of PD-1 and Tim3 as a possible therapeutic approach to restore CD8^+^ T lymphocytes’ functionality in context of melanoma [114]. The same research group identified an additional inhibitory receptor, called T cell immunoglobulin (Ig) and immunoreceptor tyrosine-based inhibition motif (ITIM) domain (TIGIT). Inhibition of this receptor together with PD-1 may counteract dysregulated T cells’ activity in a manner similar to Tim3 inhibition [115]. Extensive transcriptional profiling of the tumor infiltrate in 25 melanoma patients recently showed clonal expansion of dysfunctional CD8^+^ T cell subset. The authors highlighted the reactivity and differentiation of these cells, which are likely involved in the regulation of antitumor activity and resistance to immunotherapeutic agents, making them an attractive target for more targeted and effective immunotherapeutic treatments in melanoma [116].

B lymphocytes are the cells responsible for humoral and acquired immunity. Their main function is to produce specific antibodies against foreign antigens, but they are also involved in maintenance of immune memory [117]. In melanoma, tumor-associated B cells (TAB) account for up to 33% of TME immune cells and are involved in resistance to targeted therapy by promoting angiogenesis and chronic inflammation. In addition, the presence of B cells in the tumor infiltrate is associated with increased metastatic capacity of melanoma cells and reduced patients’ OS [118]. Recently, an interesting study analyzed the cross talk between melanoma cells and TAB and identified specific stimulating factors involved in the modulation of tumor response to different drugs. Melanoma secretes fibroblast growth factor 2 (FGF2), which actives B cells through its binding to fibroblast growth factor receptor 3 (FGFR-3) and promotes the release of insulin-like growth factor 1 (IGF-1). This factor, on tumor cells, induces proliferation and drug resistance. IGF-1, in turn, induces tumor cell proliferation and drug resistance. High levels of IGF-1 and FGFR-3 have been found in biopsies of melanoma patients treated with BRAF inhibitors in monotherapy or in combination with MEK inhibitors and IGF-1, and its receptor (IGF-1R) are associated with resistance to MAPK inhibitors [118]. However, TABs may have an opposite function in response to immunotherapy in melanoma. Indeed, a particular subtype of TABs can instead promote melanoma response to ICIs, by promoting the recruitment of CD8^+^ T cells in the tumor compartment. The authors observed that the presence of higher concentrations of these B cells, in pretreated melanoma patients, is associated with a better response to future immunotherapy treatments [119]. More recently, analysis of metastatic melanoma samples showed that the co-occurrence of tumor- associated CD8^+^ T cells and CD20^+^ B cells is associated with improved survival [120]. The formation of tertiary lymphoid structures in these CD8^+^/CD20^+^ tumors is associated with a gene signature, which predicts clinical outcomes in melanoma patients treated with ICIs. Moreover, B cell-rich melanomas displayed increased levels of transcription factor 7 (TCF7)+ naive and/or memory T cells, whereas T cells in tumors without tertiary lymphoid structures had a dysfunctional molecular phenotype. In another study, it was shown that B cell signatures are enriched in human melanoma samples from patients who responded to neoadjuvant ICI treatment [121]. B cell markers were, indeed, the most differentially expressed genes in the tumors of responders versus non responders [122]. Histological evaluation again highlighted the localization of B cells within tertiary lymphoid structures, while RNA sequencing demonstrated clonal expansion and unique functional states of B cells (switched memory B cells) in responder.

NKs are an important subclass of granular lymphocytes, involved in the recognition and elimination of virus-infected and transformed cells [123,124]. In general, cancer promotes several mechanisms that destabilize the functionality of NKs, determining immune evasion: (1) Hyperproduction of activating ligands that paradoxically block NKs’ receptors and (2) release of immunosuppressive factors, such as TGF-β and prostaglandin E [125]. Moreover, vemurafenib treatment of melanoma cells induces suppression of NKs activity in vitro, through downregulation of natural killer group 2D (NKG2D) and DNAX accessory molecule-1 (DNAM-1) activating receptors and simultaneous upregulation of MHC-I, which plays an inhibitory effect on NK cells [126].

Tregs represent a CD4^+^ T cell’s subpopulation with immunosuppressive properties [127,128]. In different cancer types, including melanoma, Tregs are able to promote immune evasion and cancer progression and are associated with poor prognosis [129,130,131]. In an analysis conducted on peripheral blood mononuclear cells (PBMCs) collected from healthy volunteers, Baumgartner and collaborators observed that melanoma evades the immune system by activation of Treg cells. Indeed, PBMCs exposed to melanoma-conditioned medium for a week presented an increase in Tregs’ induction and a major presence of IL-10 and TGF-β in the supernatant, as compared to the same PBMCs grown in control medium [132]. In BRAF-mutant melanomas, uncontrolled MAPK activation leads to an increased production of different ILs and VEGF that influence the activity of the immune system toward a protumor condition. Sumimoto and collaborators showed that in BRAF-mutant melanomas Tregs are activated and suppress the antitumor function of T lymphocytes. Moreover, pharmacological blockades or genetic manipulation of key components of MAPK pathway drastically decrease tumor production of immunosuppressive cytokines, allowing for the development of an immune microenvironment favorable to tumor suppression [133]. 

Regulation of Tregs’ differentiation and function could, therefore, be considered a valid therapeutic target for many cancers, including melanoma. Tregs are characterized by constitutive upregulation of PD-1 and CTLA-4 receptors and this condition leads to the hypothesis that Tregs could be the actual targets of ICI-based immunotherapy [134]. Unfortunately, results obtained in different studies are conflicting. Indeed, some studies have confirmed the inhibitory action of ICI on Tregs’ functionality, while others have reported opposite results that could support the hypothesis of an involvement of ICI-mediated activation of these cells in immune-resistance [135,136,137]. Analysis conducted on murine models of autoimmune pancreatitis have partly elucidated the suppressive role of PD-1 on Treg cells activity. Indeed, mice characterized by PD1-deficient Tregs showed greater immunosuppressive capacities and rapid development of autoimmune disease [135]. On the basis of these results, it can be speculated that, physiologically, the PD-1 axis plays an important role in the regulation of Tregs’ functionality and its inhibition may result in their increased activity. In vitro and in vivo experiments showed that, after treatments with nivolumab, Tregs proliferate and are functionally activated, resulting in the inhibition of antitumor activity [137]. Although with somewhat conflicting results, anti-CTLA-4 therapy would seem to bring more favorable effects on Tregs inhibition. Melanoma patients treated with ipilimumab showed a reduction in Tregs’ levels and major benefits in terms of decreased tumor growth and survival [138]. In mice models of melanoma, CTLA-4 blockade increases the intratumor effector T cells/Tregs ratio, through fragment crystallizable (Fc)-gamma receptor (FcγR)-dependent mechanism. FcγR is expressed by several immune cells, such as macrophages, neutrophils and NK cells and, therefore, TME composition may influence the response to CTLA-4 inhibitors. Melanomas presenting low concentrations of macrophages or immune cells deficient for FcγR tend to respond less to therapy [139]. 

#### 3.1.3. MDSCs 

MDSCs are immature myeloid cells, with immunosuppressive functions, associated to tumor progression, metastasis, angiogenesis, and drug resistance. Absent or present in small concentrations in physiological conditions, they are recruited by tumor cells or by inflammatory stimuli and are responsible for the production of several factors involved in tumor growth and immune evasion, such as IL-10, TGF-β, and VEGF [140,141]. In melanoma, chronic inflammation promotes MSDCs’ accumulation and activation in TME; thus, these cells are considered a possible therapeutic target in melanoma treatment [142]. A recent study identified a set of microRNAs (miRNAs) that regulate the differentiation and polarization of MDSCs in melanoma [143]. The authors found a significant association between the levels of these circulating miRNAs and reduced PFS and OS for melanoma patients treated with PD-1 and CTLA-4 inhibitors. This relationship was not reproduced in liquid biopsies of patients treated with MAPK pathway inhibitors, indicating a possible specific correlation with resistance to immunotherapy, as opposed to targeted therapy. These data suggest the possibility that combined treatments to inhibit myeloid dysfunctions could be able to overcome resistance to ICI in melanoma [143]. 

As reported by Gebhardt C. and collaborators, high levels of MDSCs in the TME are associated with ipilimumab resistance. Analysis of peripheral blood of 59 metastatic melanoma patients showed increased levels of MDSCs and their chemoattractant factors in patients poorly responsive or resistant to treatment with the CTLA-4 inhibitor. In addition, MDSCs exhibited a higher production of nitric oxide and were characterized by a higher expression of PDL-1, as compared to those isolated from responsive patients [144]. 

In melanoma, high levels of MDSCs are also associated with resistance to BRAF inhibitors. A recent preclinical study conducted in BRAF-inhibitor resistant mouse models showed that, after an initial response to treatment, these mice develop acquired resistance associated to an increase of MDSCs in TME. MAPK signaling reactivation in BRAF-resistant mice promotes the release of a complex system of stimulating cytokines, including C-C Motif Chemokine Ligand 2 (CCL2), that attract MDSCs and suppress the immune response [145].

#### 3.1.4. Tumor-Associated Macrophages (TAMs)

Macrophages are immune cells involved in phagocytosis, pro-inflammatory cytokines’ production, and specific immunity. The tumor-associated macrophages (TAMs), under the influence of cancer cells and the other microenvironment components, may be promoters or repressors of the tumorigenic process [146]. They are divided into two categories. M1-like macrophages (M1-TAMs), with antitumor activity, are important for the early stages of the inflammatory response. M2-like macrophages (M2-TAMs), predominant in TME, are correlated with tumor progression [82,147,148]. Different studies confirmed the key role of TAMs in tumor progression and have highlighted the significant correlation between the high levels of TAMs in the TME and poor prognosis for the patients [149,150]. In particular, melanoma cells, releasing miRNA-125b-5p in the microenvironment, inhibit the lysosomal acid lipase A (LIPA) and promote M2-macrophages’ phenotype and their survival [151].

TAMs induce resistance to MAPK inhibitors by favoring the expression of tumor resistance factors or through their direct paradoxical activation of the pathway, driven by BRAF inhibitors. In in vivo melanoma models, MAPK-targeted agents induce tumor necrosis factor α (TNFα) production by macrophages, which promotes NFkB pathway activation and higher MITF expression. To overcome the TNFα- and MITF-mediated resistance to MAPK inhibitors, the authors proposed a selective inhibition of NFkB pathway that, in in vitro and in vivo analyses, synergized with MEK blockade and decreased the TNFα production [152]. Moreover, TAMs suffer the paradoxical reactivation of the MAPK pathway under the influence of BRAF inhibitors, with increased production of pro-angiogenic factors, such as VEGF and IL-8 and resistance to treatments [153]. 

TAMs express high levels of V-domain Ig suppressor of T cell activation (VISTA), another negative immune checkpoint, which is correlated with resistance to immunotherapy [154,155]. VISTA, associated with a significant decrease of survival in primary melanomas, in vivo, promotes a protumoral microenvironment mediated by upregulation of Tregs’ levels and PDL-1 expression on macrophages’ surface [155,156,157,158].

The switch of TAMs to an antitumor phenotype is considered an alternative approach to evade TAM-mediated resistance and to reconstitute the response to PD-1 axis inhibitors. TAMs’ transformation process, mediated by STAT6 inactivation and NFkB phosphorylation, results in an increase of IL-12 production by TAMs and a reduction in inhibitory cytokine levels, such as IL-10 and C-C motif chemokine 22 (CCL22) [159]. TAMs induce immunotherapy resistance also by inhibiting the recruitment of CD8^+^ T lymphocytes in the tumor site. Indeed, TAMs, by stable and durable interactions with T cells, lead to the maintenance of an immunosuppressive microenvironment not responsive to the inhibitory activity of anti-PD1 molecules [160]. 

#### 3.1.5. DCs

DCs are immune cells derived from myeloid precursors and are implicated in recognition and capture of antigens considered "foreign", such as pathogens or cancer cells. They are antigen presenting cells (APCs) and interact with T lymphocytes through MHC present on their surface. DCs are involved in the production of cytokines and chemokines with anti- or pro-inflammatory function according to the stimuli received from the surrounding environment [161,162,163]. 

Unlike other immune cells, the involvement of DC in resistance to targeted and immunotherapy is mainly associated with the absence in tumor infiltrate of this type of cell. Melanoma is able to elude the complex mechanism of T cell activation by influencing DCs’ maturation. Tumor cells produce inhibitory cytokines such as IL-8, IL-10, and VEGF and create an unfavorable environment for DCs’ maturation. This condition negatively affects the DCs’ ability to present antigen to T cells and, therefore, determines a reduced immune response [164]. Moreover, López González and collaborators demonstrated that, if inhibition of glycogen synthase kinase 3 beta (GSK3β) obstructs DC differentiation, a constitutively active GSK3β overcomes the IL-10 inhibition, leading to DC maturation [165]. In addition, melanoma promotes the switch of myeloid cells through immuno-suppressive macrophage-like cells rather than DCs [166]. The use of oncolytic virus (i.e., ORCA-010) could stimulate a specific differentiation of DCs and T cell priming by producing tumor-associated neo-antigen in order to increase the response to ICIs [167]. The therapeutic potential of DC vaccines was, recently, supported in an interesting preclinical work by Zhou and collaborators. The authors produced in vitro CD103+ murine and evaluated its activity in murine models of melanoma and osteosarcoma. CD103+ stimulated a favorable environment to the action of T lymphocytes, resulting in a reduced primary and metastatic tumor growth [168]. Further and recent results have also been obtained by direct DC targeting with molecular inhibitors. A recent study demonstrated that dasatinib (Tyrosine Kinase inhibitor) induces the activation of allogenic T cells by impairing the phosphorylation and metabolism of tryptophan induced by Indoleamine-2,3-dioxygenase (IDO), one of the most important intermediary cancer tolerants [169]. In addition, the same RAF kinase inhibitors could induce acquired resistance by influencing the differentiation and activation of DCs. Preclinical experiments, carried out on human and mouse DC cells, have detected a reduced or lack of DCs’ ability to recruit T cells after treatment with RAF kinase inhibitors. These experiments, therefore, open possible new therapeutic scenarios, not only in melanoma, considering the negative effects of pan-RAF inhibitors on the immune response modulation [170].

### 3.2. ECM

ECM is an intricate network of proteins, proteoglycans, and glycoconjugates produced by TME cells and involved in the adhesion and support of the cellular compartment [171]. In different cancer contexts, the tumor matrix, creating a physical barrier, blocks drugs and inhibits their action. The mutual interactions between tumor and stroma cells induce rearrangements of the matrix architecture; this remodeling promotes tumor progression and modulates response to treatment [81].

Fibronectin, produced by CAFs, represents the major component of ECM and seems to play a key role in a decrease of sensitivity to therapies in different solid tumors [172,173]. BRAF-mutant and PTEN-loss melanomas show, after an initial response to treatments, the development of resistance to BRAF inhibitors mediated by reactivation of MAPK and PI3K pathways. In this molecular background, drug resistance seems to be mediated also by the protective effect of fibronectin, upregulated by BRAF inhibition [174]. Phosphoproteomic analysis conducted on BRAF-mutant melanoma cell lines showed, after treatment with vemurafenib or after BRAF gene silencing, an increased expression of fibronectin, only in PTEN-loss contexts. This evidence and further experiments conducted in cells genetically manipulated for PTEN have promoted the idea that regulation of fibronectin expression is associated with PTEN. Moreover, clinical data confirmed the higher expression of fibronectin in tissue of melanoma patients with PTEN loss [174]. The interaction between fibronectin and its receptor integrin α5β1 leads to AKT phosphorylation and decreases the apoptotic capacity of melanoma cells as a result of higher activity of myeloid leukemia cell protein 1 (MCL-1). Therefore, BRAF inhibition promotes a remodeling of melanoma microenvironment, by which the tumor cells escape to pharmacological blockade. In the molecular contexts analyzed, the study proposed BRAF/PI3K inhibitor combinations as an alternative to overcome the development of secondary resistance to BRAF-targeted agents [174]. 

Integrins are transmembrane receptors that physically regulate the interaction between cells and the ECM components and promote the development of intracellular signals. To date, 24 receptors have been identified, consisting of 18 subunits of α and eight of β, and several integrins are associated with melanoma progression and metastasis [175,176]. Integrin α5β1 is the most important fibronectin receptor and their interaction modulates several cellular processes, such as adhesion, migration, and cellular differentiation [173,177]. An interesting work of intravital imaging of BRAF-mutant melanoma cells showed that, in co-culture systems, the treatment with BRAF inhibitors determines reactivation of MAPK pathway in areas with high stromal density; this condition is influenced by matrix remodeling associated to integrin β1/FAK/Src signaling [95]. Under the influence of treatment, the tumor fibroblasts suffer a paradoxical activation of ERK, resulting in higher fibronectin production and interaction with its receptor on tumor cells. Integrin α5β1 promotes in melanoma cells the FAK-mediated ERK reactivation and resistance to BRAF inhibitors [95]. Integrin αvβ3 is another receptor involved, in melanoma context, in immunotherapy resistance through its regulation of the PDL-1 expression [178]. In in vitro and in vivo melanoma models, several evidences showed that integrin αvβ3 promotes the expression of PDL-1 through activation of STAT1 [179]. 

The matrix remodeling is influenced by a family of MMPs, enzymes involved in hydrolysis of the other proteins and in mobility of tumor cells. In melanoma, several MMPs are involved in the different aspects of tumorigenic process, such as drug resistance [180]. Vemurafenib treatment in melanoma-resistant cells supports the paradoxical reactivation of ERK, a significant increase of IL-8 levels, and activation of MMPs, especially MMP2. This condition promotes the matrix disorganization, tumor motility, and immune evasion [181]. 

## 4. Conclusions

Currently, targeted therapy and immunotherapy represent a consolidated reality for the treatment of many aggressive tumors, such as metastatic melanoma. However, like previous therapeutic approaches, they are not exempt from the problem of innate and acquired resistance, which determines the failure of treatment in an important cohort of patients. Until a few years ago, scientific research was focused on the genetic and somatic changes by which cancer cells evaded drug inhibition. However, the new evidences on TME have revealed the existence of an intricate network of interconnections between the tumor and the surrounding microenvironment that massively influences all phases of the tumorigenic process. Through direct contact or release of soluble factors, the components of TME continuously influence tumor cells’ activity modulating the drug response and therapeutic outcome. For these reasons, the research activities have focused on identifying the mechanisms and phenomena that TME implements to induce resistance to treatments. Based on the molecular mechanisms described in this review, it is evident that CAFs play a key role in the development of resistance to targeted therapy, especially through the production of many paracrine factors. Instead, the recruitment of immunosuppressive components, such as Tregs and MDSCs, in the TME is the primary mechanism of resistance to immunotherapy.

## Abbreviations

AKT	Protein kinase B
APC	Antigen presenting cell
APE1	Apurinic/apyrimidinic endonuclease 1
BAD	BCL2 associated agonist of cell death
BRAF	v-Raf murine sarcoma viral oncogene homolog B
CAFs	Cancer-associated fibroblasts
CAR-T	Chimeric antigen receptor T-cells
CCL2	C-C Motif Chemokine Ligand 2
CCL-22	C-C motif chemokine 22
CD	Cluster differentiation
CRAF	v-raf1 murine leukemia viral oncogene homolog 1
CTL	Cytotoxic lymphocytes
CTLA-4	cytotoxic T-lymphocyte-associated protein 4
CXCL-2	CXC chemokine ligand 2
CXCL5	CXC motif chemokine 5
DCs	Dendritic cells
DNAM-1	DNAX accessory molecule-1
ECM	Extracellular matrix
EMT	Epithelial mesenchymal transition
ErbB3	V-erb-b2 avian erythroblastic leukemia viral oncogene homolog3
ERK	Extracellular signal-regulated kinase
FAK	Focal adhesion kinase
FAP	Fibroblast activation protein
FcγR	Fragment crystallizable-gamma receptor
FDA	Food and Drug Administration
FGF 2-19	Fibroblast growth factor 2-19
FGFR-3	Fibroblast growth factor receptor 3
FSP1	Fibroblast specific protein 1
GSK3β	Glycogen synthase kinase 3 beta
Gp	Glycoprotein
HER3	V-erb-b2 avian erythroblastic leukemia viral oncogene homolog 3
HGF	Hepatocyte growth factor
ICIs	Immune checkpoint inhibitors
IDO	Indoleamine-2,3-dioxygenase
Ig	Immunoglobulin
IGF-1	Insulin-like growth factor 1
IGF-1R	Insulin-like growth factor receptor 1
IL	Interleukin
ITIM	Immunoreceptor tyrosine-based inhibition motif
KIT	v-kit Hardy–Zuckerman 4 feline sarcoma viral oncogene homolog
LIPA	Lysosomal acid lipase A
MAPK	Mitogen-activated protein kinase
MCL-1	Myeloid leukemia cell protein 1
MCH-I	Major histocompatibility complex 1
MDSCs	Myeloid-derived suppressor cells
MEK	Mitogen-activated protein kinase kinase
MET	Mesenchymal epithelial transition receptor
MHC	Major histocompatibility complex
miRNA	microRNA
MITF	Microphthalmia-associated transcription factor
MMPs	Matrix metalloproteinases
NFkB	Nuclear factor kappa-light-chain-enhancer of activated B cells
NGS	Next-generation sequencing
NK	Natural killer
NKG2D	Natural killer group 2D
NRAS	Neuroblastoma RAS viral oncogene homolog
NRG-1	Neuregulin 1
ORR	Objective response rate
OS	Overall survival
PBMC	Peripheral blood mononuclear cells
PD-1	Programmed cell death protein 1
PDGFR	Platelet-derived growth factor receptor
PDL-1/2	Programmed death ligands 1/2
PI3K	Phosphatidyl Inositol 3-kinase
PFS	Progression-free survival
PTEN	Phosphatase and tensin homolog on chromosome 10
ROS	Reactive oxygen species
RTK	Tyrosine kinase receptor
sFRP2	Secreted frizzled-related protein 2
STAT	Signal transducer and activator of transcription
SRC	V-src sarcoma (Schmidt–Ruppin A-2) viral oncogene homolog avian
TAB	Tumor-associated B cells
TAMs	Tumor-associated macrophages
TCF7	Transcription factor 7
TGF-β	Transforming growth factor β
TIGIT	T cell Ig and ITIM domain
TIL	Tumor-infiltrating lymphocytes
Tim3	Immunoglobulin and mucin domain-containing molecule 3
TME	Tumor microenvironment
TNFα	Tumor necrosis factor α
TRAF6	Tumor necrosis factor receptor-associated factor 6
Tregs	Regulatory T cells
VEGF	Vascular endothelial growth factor
VISTA	V-domain Ig suppressor of T cell activation
Wnt	wingless type MMTV integration site family member
α-SMA	α smooth muscle actin
